# Method for Essential Protein Prediction Based on a Novel Weighted Protein-Domain Interaction Network

**DOI:** 10.3389/fgene.2021.645932

**Published:** 2021-03-17

**Authors:** Zixuan Meng, Linai Kuang, Zhiping Chen, Zhen Zhang, Yihong Tan, Xueyong Li, Lei Wang

**Affiliations:** ^1^College of Computer, Xiangtan University, Xiangtan, China; ^2^College of Computer Engineering & Applied Mathematics, Changsha University, Changsha, China

**Keywords:** essential proteins, protein-protein interaction network, computational model, domain-domain interaction network, protein-domain interaction network

## Abstract

In recent years a number of calculative models based on protein-protein interaction (PPI) networks have been proposed successively. However, due to false positives, false negatives, and the incompleteness of PPI networks, there are still many challenges affecting the design of computational models with satisfactory predictive accuracy when inferring key proteins. This study proposes a prediction model called WPDINM for detecting key proteins based on a novel weighted protein-domain interaction (PDI) network. In WPDINM, a weighted PPI network is constructed first by combining the gene expression data of proteins with topological information extracted from the original PPI network. Simultaneously, a weighted domain-domain interaction (DDI) network is constructed based on the original PDI network. Next, through integrating the newly obtained weighted PPI network and weighted DDI network with the original PDI network, a weighted PDI network is further constructed. Then, based on topological features and biological information, including the subcellular localization and orthologous information of proteins, a novel PageRank-based iterative algorithm is designed and implemented on the newly constructed weighted PDI network to estimate the criticality of proteins. Finally, to assess the prediction performance of WPDINM, we compared it with 12 kinds of competitive measures. Experimental results show that WPDINM can achieve a predictive accuracy rate of 90.19, 81.96, 70.72, 62.04, 55.83, and 51.13% in the top 1%, top 5%, top 10%, top 15%, top 20%, and top 25% separately, which exceeds the prediction accuracy achieved by traditional state-of-the-art competing measures. Owing to the satisfactory identification effect, the WPDINM measure may contribute to the further development of key protein identification.

## Introduction

Accumulating evidence indicates that proteins have a tremendous impact on almost all life activities. Essential proteins cannot only maintain normal biological processes but also ensure the integrity of cell functions. With the development of biotechnology (Lu et al., 2019, 2020), more and more essential proteins have been discovered by biological experiments in recent years. However, because biological experiments are quite costly and time-consuming, an increasing number of computational models have been proposed to identify essential proteins based on the topological features of PPI networks. For instance, based on the rule of centrality-lethality (Jeong et al., 2001), researchers have proposed a series of prediction models, which have been designed successively to infer potential critical proteins. These include Information Centrality (IC) (Stephenson and Zelen, 1989), Degree Centrality (DC) (Hahn and Kern, 2004), Subgraph Centrality (SC) (Ernesto and Rodriguez-Velazquez, 2005), Closeness Centrality (CC) (Wuchty and Stadler, 2003), Betweenness Centrality (BC) (Jop et al., 2005), Neighbor Centrality (NC) (Wang et al., 2012), and local average connectivity (LAC) (Li et al., 2015). Wang et al. (2011) designed a predictive model named SoECC by combining the features of edges and nodes and taking advantage of the edge clustering coefficient effectively. Lin et al. (2008) introduced two kinds of prediction models such as the Maximum Neighborhood Component (MNC) and the Density of Maximum Neighborhood Component (DMNC) to infer essential proteins, respectively. However, these prediction models cannot achieve high identification accuracy owing to the incompleteness of current PPI networks (Chen and Yuan, 2006).

Hence, to address this problem, some different methods based on both biological information on proteins and the topological properties of PPI networks have been proposed to detect essential proteins. For example, Li et al. (2012) proposed a calculation method called Pec by uniting the gene expression data with the centrality-lethality rule to identify key proteins from PPI networks. Zhang et al. (2013) presented a method based on integrating the topological features of PPI networks with the co-expressions of proteins. Peng et al. (2012) designed a prediction method called ION based on topological features extracted from the PPI network and the orthologous information of proteins. Additionally, inspired by the model of Degree Centrality, Tang et al. (2014) developed an identification model for predicting essential proteins by combining the Person correlation coefficient (PCC) and the edge clustering coefficient (ECC) with the gene expression data of proteins. Kim (2012) proposed a method for predicting key proteins by implementing a machine learning algorithm on both Gene Ontology and topological information of PPI networks. Luo et al. (2015) developed a computational model by integrating the local interaction density with protein complexes to detect key proteins. Li et al. (2016) designed a method for identifying essential proteins by adopting the subcellular localization and orthologous information. Luo and Kuang (2014) proposed a prediction model called CDLC to detect essential proteins by employing the dynamic local average connectivity and in-degree of proteins in complexes. Zhang et al. (2018) introduced a calculative algorithm named TEO for inferring essential proteins by integrating gene ontology annotation information and the gene expression data with PPI networks. Zhong et al. (2013) designed a learning algorithm to predict essential proteins by combining the biological information of proteins with PPI networks. Shang et al. (2016) introduced a strategy to detect essential proteins through integrating the RNA-Seq dataset and biological information of proteins with dynamic PPI networks. Zhang et al. (2016) introduced a prediction measure named PINs for identifying essential proteins based on gene expression profiles and PPI networks through integrating five approaches including the DC, BC, SC, CC, and the eigenvector centrality (EC) (Bonacich, 1987).

This study proposes a novel prediction model called WPDINM that can be used to detect key proteins by combing a weighted protein-domain interaction (PDI) network with the biological information containing the subcellular localization and orthologous information of proteins. WPDINM is based on the original PPI network and the original PDI network, obtained by known protein-protein interactions (PPIs) and known protein-domain associations that have been downloaded from benchmark databases. In this prediction model, a weighted PPI network and a weighted domain-domain interaction (DDI) network are established first, based on the gene expression data of proteins and the topological information of the original networks respectively. Then, a weighted PDI network is constructed by combining these two newly constructed weighted networks. Next, based on the weighted PDI network, initial scores are assigned to proteins based on the biological information of proteins such as the subcellular localization and orthologous information of proteins, and a novel iterative method is implemented to estimate the criticality of proteins.

Different from traditional prediction models, in WPDINM, the Discrete Fourier transform (DFT) is applied to the gene expression profiles of proteins to calculate the weight between proteins, which can translate gene expression profiles from the time domain to frequency domain effectively. A novel weighted PDI network is then constructed by integrating a weighted DDI network and weighted PPI network. Moreover, by taking into account the associations between proteins, a new directed distribution network is designed to calculate the rankings of proteins iteratively, based on the weighted PDI network. Finally, to evaluate the prediction performance of WPDINM, the WPDINM is compared with other competitive measures such as SC (Ernesto and Rodriguez-Velazquez, 2005), DC (Hahn and Kern, 2004), IC (Stephenson and Zelen, 1989), CC (Wuchty and Stadler, 2003), BC (Jop et al., 2005), NC (Wang et al., 2012), EC (Bonacich, 1987), Pec (Li et al., 2012), CoEWC (Zhang et al., 2013), TEGS (Zhang et al., 2019), ION (Peng et al., 2012), and POEM (Zhao et al., 2014). Experimental results indicate that WPDINM can achieve better prediction accuracies than competing prediction models, achieving 90.19, 81.96, 70.72, 62.04, 55.83, and 51.13% in the top 1%, top 5%, top 10%, top 15%, top 20%, and top 25% of predicted proteins separately.

## Materials and Methods

### Experimental Data

To construct original PPI networks, we first download known PPIs from three different databases including the DIP database (Xenarios et al., 2002), the Gavin database (Gavin et al., 2006), and the Krogan database (Krogan et al., 2006), respectively. After removing duplicated interactions, we finally obtain three different datasets such as the DIP-based dataset, consisting of 24,743 known PPIs between 5,093 proteins, the Krogan-based dataset, consisting of 14,317 known PPIs between 3,672 proteins, and the Gavin-based dataset consisting of 7,669 known PPIs between 1,855 proteins. Next, we further download known domains from the Pfam database (Bateman et al., 2004), and after preprocessing, obtain a dataset consisting of 1,107 different domains. Based on these three kinds of datasets obtained from the DIP database, the Gavin database, and the Krogan database, we finally construct three kinds of original PPI networks and corresponding matrices with dimensions of 5,093 = 1,107, 1,855 = 1,107, and 3,672 = 1,107 separately. The gene expression data is provided by Tu et al. (2005), which consists of 6,776 gene expression sequences with a length of 36.

In order to obtain the initial scores of proteins, we download the subcellular localization data from the COMPART-MENTS databases (Binder et al., 2014). As a result, we obtain a dataset consisting of 11 kinds of subcellular localizations, including the Extracellular, Peroxisome, Nucleus, Plasma, Endosome, Mitochondrion, Vacuole, Cytosol, Golgi, Cytoskeleton Endoplasmic, that are intimately linked with downloaded known key proteins. We also download the orthologous information of proteins from the InParanoid database (Gabriel et al., 2010). Furthermore, the set of essential proteins existing in Saccharomyces cerevisiae is was downloaded from four different databases including DEG (Zhang and Lin, 2009), MIPS (Mewes et al., 2006), SGD (Cherry et al., 1998), and SGDP (Saccharomyces Genome Deletion Project, 2012).

**FIGURE 1:**
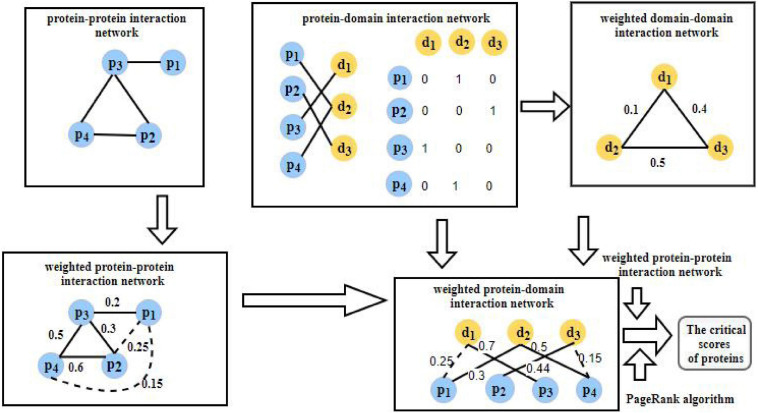
Flowchart of WPDINM.

As shown in Figure 1, the flowchart of WPDINM consists of the following four major steps:

**Step 1:** Firstly, based on known PPIs downloaded from any given benchmark database, an original PPI network is obtained. Then, a weighted PPI network is further constructed by implementing the DFT method on the gene expression data of proteins and extracting topological features from the original PPI network.

**Step 2:** Based on known PPIs and known protein-domain interactions (PDIs) downloaded from given benchmark databases, a weighted DDI network is then constructed. Thereafter, a weighted PDI network is further established by integrating the weighted DDI network with the weighted PPI network.

**Step 3:** Then, by combining the weighted PDI network with biological information, including the orthologous information and subcellular information of proteins, each protein in the weighted PDI network is assigned an initial score.

**Step 4:** Finally, a novel prediction method based on the Page Rank algorithm is designed and applied on the weighted PDI network to compute the final scores of criticality for all proteins iteratively.

### Construction of the Weighted PPI Network

In this section, based on the datasets consisting of known PPIs downloaded from three different databases, including the DIP database (Xenarios et al., 2002), the Gavin database (Gavin et al., 2006), and the Krogan database (Krogan et al., 2006), respectively, we construct three original PPI networks simultaneously. For convenience, let *OppiN* = {*N*_*P*_,*E*_*P*_} represent a newly constructed original PPI network, where *N*_*P*_ = {*p*_1_,*p*_2_….*p*_*O*_} is the set of protein nodes in *OppiN* and *E_P_* is the set of edges between protein nodes in *OppiN*. Here, for two given proteins *p_i_* and *p_j_*in *N_P_*, there is an edge *ed*(*p*_*i*_,*p*_*j*_) between them in *E_P_*, if and only if there is a known interaction between these two proteins. Based on the original PPI network *OppiN*, we can further obtain an *O × O* dimensional adjacency matrix*OppiM* as follows: for any two protein *p_i_* and *p_j_* in *N_P_*, there is *OppiM*(*i*,*j*) = 1, only if there is a known interaction between *p_i_*and *p_j_*, otherwise there is *OppiM*(*i*,*j*) = 0.

Next, based on *OppiN*, for any given protein *p* with a known gene expression sequence in *N_P_*, let *Gep*(*p*) = < *Gep*(*p*,1),*Gep*(*p*,2),…,*Gep*(*p*,*M*) > represent the gene expression sequence of *p*, where *Gep*(*p*,*t*) is the degree of gene expression at *t*^*th*^ time. As *Gep*(*p*) is a time sequence with the length of *M*, then we can adopt the DFT method to convert it from time domains to frequency domains, since while *N ≥ M*, the *N*-point Discrete Fourier can transform a 1 × *M* dimensional time series vector *Gep*(*p*)to a 1 × *N* dimensional spectrum vector *DF*(*p*) = < |*DGep*(0)|,|*DGep*(1)|,…|*DGep*(*N*−1)| > as follows:

(1) $$DGep\left(t\right)=\sum_{\text{y}=1}^{M}Gep\left(p,y\right)*W_{M}^{t\,y},\text{where}t=0,1,\ldots ..N-1$$

(2) $$W_{M}=e^{-i\,\frac{2\,\pi }{M}}$$

Thereafter, through combining the above formulas with the Gaussian kernel interaction profiles, for any two given proteins *p_i_* and *p_j_*with gene expression sequences in *N_P_*, we can estimate the probability of association between them by calculating the spectra similarity as follows:

(3) $$G\,K\,\left(p_{i},p_{j}\right)=\exp\left(-\alpha_{\text{p}}\,{||D\,F\,\left(p_{i}\right)-D\,F\,\left(p_{j}\right)||}^{2}\right)$$

Here, α_*p*_ is the adjustment coefficient for the kernel bandwidth, which is defined as follows:

(4) $$\alpha_{p}=\frac{\alpha_{p}^{\prime }}{\frac{1}{\left|P_{\text{Gep}}\right|}\,\sum_{k=1}^{\left|P_{\text{Gep}}\right|}{||D\,F\,\left(p_{k}\right)||}^{2}}$$

In the above formula (4), *P*_*Gep*_ is the set of proteins with gene expression sequences in *OppiN*.

Additionally, for any two given proteins *p_i_* and *p_j_* without gene expression sequences in *OppiN*, we adopt the topological features extracted from the original PPI network *OppiN* to calculate the possibility of an association between them. Thus, the weight between *p_i_* and *p_j_* can be calculated as follows:

(5) $$T\,F\,P\,\left(p_{i},p_{j}\right)=\frac{\left|C\,o\,m\,\left(p_{i},p_{j}\right)\right|+1}{\left(\left|N\,p\,\left(p_{i}\right)\right|+1\right)*\left(\left|N\,p\,\left(p_{j}\right)\right|+1\right)}$$

Here, *Np*(*p*_*i*_) and *Np*(*p*_*j*_) denote the set of neighboring protein nodes of *p_i_* and *p_j_* in *OppiN* separately, *Com*(*p*_*i*_,*p*_*j*_) represents the set of common neighbors between *p_i_* and *p_j_* in *OppiN*, and |*X*| means the number of different elements in the set *X*.

Integrating formula (3) and formula (5) for any two given proteins *p_i_* and *p_j_* in *OppiN*, we can calculate the possibility of an association between them, b, as follows:

(6) $$P\,A\,\left(p_{i},p_{j}\right)=\left\{ \begin{array}{c} G\,K\,\left(p_{i},p_{j}\right):i\,f\,p_{i}\,\mathrm{and}\,p_{j\,h\,a\,v\,e\,g\,e\,n\,e\,e\,x\,p\,r\,e\,s\,s\,i\,o\,n\,s\,e\,q\,u\,e\,n\,c\,e\,s}\\ TFP\left(p_{i},p_{j}\right):\;\mathrm{Otherw}\text{ise} \end{array}\right.$$

Based on the above formulas (6), a weighted PPI network *WppiN* can be constructed according to the following *O × O* dimensional matrix *WppiM*:

(7) $$W\,p\,p\,i\,M\,\left(p_{i},p_{j}\right)=\left\{ \begin{array}{c} \beta *P\,A\,\left(p_{i},p_{j}\right)+\left(1-\beta \right)*O\,p\,p\,i\,M\,\left(p_{i},p_{j}\right)\,\mathrm{if}\,O\,p\,p\,i\,M\,\left(p_{i},p_{j}\right)=1\\ T\,F\,P\,\left(p_{i},p_{j}\right)*G\,K\,\left(p_{i},p_{j}\right)\;\mathrm{if}\,O\,p\,p\,i\,M\,\left(p_{i},p_{j}\right)=0 \end{array}\right.$$

In the above formula (7), β is the scaling parameter with a value from 0 to 1.

### Construction of the Original PDI Network

In this section, based on the dataset consisting of known PDIs downloaded from the Pfam database (Bateman et al., 2004), we construct an original PDI network *OpdiN* = {*N*_*PD*_,*E*_*PD*_}, where *N*_*PD*_ = *N*_*P*_∪*N*_*D*_, *N*_*D*_ = {*d*_1_,*d*_2_…*d*_*Q*_} is the set of domain nodes in *OpdiN*, and *E*_*PD*_ is the set of edges between protein nodes in *N_P_* and domain nodes in *N_D*. Here, for a given protein *p_i_* and a given domain *d_j_* in *N*_*PD*_, there is an edge between them in *E*_*PD*_, only if there is *p_i_* belonging to *d_j_*. Based on the original PDI network *OpdiN*, we can further obtain an *O × Q* dimensional adjacency matrix *OpdiM* as follows: for a given protein node *p_i_* and a given domain node *d_j_* in *N*_*PD*_, there is *OpdiM*(*i*,*j*) = 1, if and only if there is *p_i_* belonging to *d_j_*, otherwise, there is *OpdiM*(*i*,*j*) = 0.

### Construction of the Weighted DDI Network

For any two given domains *d_i_* and *d_j_* in *OpdiN*, in this section, we further obtain a *Q × Q* dimensional matrix *WddiM* by adopting the Gaussian kernel interaction profiles to estimate the association between *d_i_* and *d_j_* as follows:

(8) $$W\,d\,d\,i\,M\,\left(d_{i},d_{j}\right)=\exp\left(-\delta_{d}\,{||I\,P_{d}\,\left(d_{i}\right)-I\,P_{d}\,\left(d_{j}\right)||}^{2}\right)$$

Here, *IP*_*d*_(*d*_*l*_) denotes the vector at the *l*^*th*^ column of the matrix *OpdiM*, and δ_*d*_ is an adjustment coefficient for the kernel bandwidth based on the new bandwidth parameter $\delta_{d}^{\prime }$, which is defined as follows:

(9) $$\delta_{d}=\frac{\delta_{d}^{\prime }}{\frac{1}{Q}\,\sum_{k=1}^{Q}{||I\,P_{d}\,\left(d_{k}\right)||}^{2}}$$

Based on the above formula (8), it is easy to construct a weighted DDI network *WddiN*.

### Construction of the Weighted PDI Network

In this section, through combining the weighted PPI network *WppiN* and original PDI network *OpdiN* with the weighted DDI network *WddiN*, we calculate two *O* × *Q* dimensional matrices *WpdiM* and *WdpiM* as follows:

(10) $$W\,p\,d\,M\,\left(t_{i},t_{j}\right)=\left\{ \begin{array}{c} WppiM\left(t_{i},t_{j}\right):\;ift_{i}\in N_{P}\mathrm{and}t_{j}\in N_{P}\\ WddiM\left(t_{i},t_{j}\right):\;elseift_{i}\in N_{D}\text{and}t_{j}\in N_{D}\\ \frac{\sum_{k=1}^{O}W\,p\,p\,i\,M\,\left(t_{i},t_{k}\right)\,O\,p\,d\,i\,M\,\left(t_{k},t_{j}\right)}{\sum_{k=1}^{O}W\,p\,p\,i\,M\,\left(t_{i},t_{k}\right)}:\;elseift_{i}\in N_{P}\mathrm{and}t_{j}\in N_{D} \end{array}\right.$$

(11) $$W\,d\,p\,M\,\left(t_{i},t_{j}\right)=\left\{ \begin{array}{c} WppiM\left(t_{i},t_{j}\right):\;ift_{i}\in N_{P}\text{and}t_{j}\in N_{P}\\ WddiM\left(t_{i},t_{j}\right):\;elseift_{i}\in N_{D}\mathrm{and}t_{j}\in N_{D}\\ \frac{\sum_{k=1}^{Q}O\,p\,d\,i\,M\,\left(t_{i},t_{k}\right)\,W\,d\,d\,i\,M\,\left(t_{k},t_{j}\right)}{\sum_{k=1}^{Q}W\,d\,d\,i\,M\,\left(t_{k},t_{j}\right)}:e\,l\,s\,e\,i\,f\,t_{i}\in N_{P}\,\mathrm{and}\,t_{j}\in N_{D} \end{array}\right.$$

Thereafter, for any two given nodes *t*_*i*_*andt*_*j*_ in *OpdiN*, we can obtain a new *O* × *Q* dimensional matrix WPDIM as follows:

(12) $$W\,p\,d\,i\,M\,\left(t_{i},t_{j}\right)=\frac{W\,p\,d\,M\,\left(t_{i},t_{j}\right)+W\,d\,p\,M\,\left(t_{i},t_{j}\right)}{2}$$

According to the above formula (12), it is easy to construct a weighted PDI network *WpdiN.*

### Calculation of the Initial Scores of Proteins

First, based on the weighted PDI network *WpdiN*, for a given protein *p_i_* and a given domain *d_j_* in *N*_*PD*_, we can obtain a *Q* × *O* dimensional allocation probability matrix *APM* as follows:

(13) $$A\,P\,M\,\left(d_{j},p_{i}\right)=\frac{W\,p\,d\,i\,M\,\left(d_{j},p_{i}\right)}{\sum_{p_{k}\in d_{j}}WpdiM\left(d_{j},p_{k}\right))}$$

Next, for simplicity, let the initial score vector for all domains in *WpdiN* be *S*_*d*_ = < 1,1,1 > ^*T*^, we assign an initial score of 1 to each domain in *WpdiN*, then based on the allocation matrix *APM*, we can distribute the initial scores of domains to all proteins in *WpdiN* in the following way:

(14) $$P\,S\,D=A\,P\,M^{T}*S_{d}$$

*PSD* is an *O* dimensional vector, and *PSD*(*i*) denotes the score, which is the *i*^*th*^ protein node *p*_*i*_ obtained from all domain nodes in *WpdiN*.

To calculate the score of the subcellular localization feature, let *N*_*SL*_ represent the number of all subcellular localizations, and *N*_*SL*_(*j*) denote the number of proteins related to the *j*^*th*^ subcellular localization. The *s*_*avg* means the average sum of the protein associated with subcellular localization. Then, the score of *j*^*th*^ subcellular localization can be computed as follows:

(15) $$S_{S\,L}\,\left(j\right)=\frac{N_{S\,L}\,\left(j\right)}{S\,\_\,a\,v\,g}$$

Where:

(16) $$S\,\_\,a\,v\,g=\frac{\sum_{j=1}^{N_{S\,L}}N_{S\,L}\,\left(j\right)}{N_{S\,L}}$$

Hence, for any given protein *p_i_*, its subcellular localization feature score can be calculated as follows:

(17) $$F\,S_{S\,L}\,\left(p_{i}\right)=\sum_{j\in S\,L\,\left(p_{i}\right)}S_{S\,L}\,\left(j\right)$$

Where *SL*(*p*_*i*_) is the set of subcellular localization related to the protein *p_i_*.

In addition, because triangles have the characteristic of stability, we further adopt the topological feature of triangles extracted from the *OppiN* to calculate at biological feature score for each protein *p_i_*. Here, for a given protein *p_i_*, its set of neighbor nodes is represented as *Np*(*p*_*i*_), then there is:

(18) $$N\,p\,\left(p_{i}\right)=\left\{q|e\,d\,\left(p_{i},q\right)\in E_{d}\right\}$$

Therefore, the triangles for protein *p_i_* is computed as follows:

(19) $$T\,R\,I\,\left(p_{i},q\right)=\left\{ \begin{array}{c} \left|N\,p\,\left(p_{i}\right)\cap N\,p\,\left(q\right)\right|+1\;\mathrm{if}\,e\,d\,\left(p_{i},q\right)\in E_{P}\\ 1\;\mathrm{if}\,e\,d\,\left(p_{i},q\right)\notin E_{p} \end{array}\right.$$

(20) $$T\,R\,I\,\left({\text{p}}_{\text{i}}\right)=\sum_{q\in N\,p\,\left(p_{i}\right)}T\,R\,I\,\left(p_{i},q\right)$$

(21) $$A\,v\,g_{T\,R\,I}\,\left(p_{i}\right)=\frac{T\,R\,I\,\left(p_{i}\right)}{\left|\mathrm{Np}\,\left(p_{i}\right)\right|}$$

Where the *TRI*(*p*_*i*_) is the set of triangles related to the protein *p_i_* and |*Np*(*p*_*i*_)| represents the degree of the protein *p_i_*. According to the above calculated triangle numbers for each protein, we compute the triangle feature score for *p_i_*;

(22) $$F\,S_{T\,R\,I}\,\left(p_{i}\right)=\frac{A\,v\,g_{T\,R\,I}\,\left(p_{i}\right)}{{\text{max}}_{1\leq \text{j}\leq \text{O}}\,A\,v\,g_{T\,R\,I}\,\left(p_{j}\right)}$$

Based on the orthologous information obtained from the InPaianoid database (Gabriel et al., 2010), for any given protein *p_i_*, let *f*_*oth*_(*p*_*i*_) be its score of orthologous information, then we can calculate an orthologous feature score for *p_i_* as follows:

(23) $$F\,S_{O\,R\,T}\,\left(p_{i}\right)=\frac{f_{o\,t\,h}\,\left(p_{i}\right)}{{\text{max}}_{1\leq \text{j}\leq O}\,f_{o\,t\,h}\,\left(p_{j}\right)}$$

Based on the above formulas (14)∼(18), for any given protein *p_i_*, we can obtain its feature score as follows:

(24) $$F\,S\,\left(p_{i}\right)=\varphi *F\,S_{S\,L}\,\left(p_{i}\right)+\theta *F\,S_{T\,R\,I}\,\left(p_{i}\right)+\tau *F\,S_{O\,R\,T}\,\left(p_{i}\right)$$

Where φ,θ and τ are proportion parameters, which are used to adjust the ratio of feature score for proteins and satisfy φ+θ+τ=1.

Finally, according to the above formula (13) and formula (19), for any given protein *p_i_*, we can obtain its initial score as follows:

(25) $$S_{0}\,\left(p_{i}\right)=\omega *P\,S\,D\,\left(p_{i}\right)+\left(1-\omega \right)*F\,S\,\left(p_{i}\right)$$

Here, ω is a proportion parameter.

### Construction of the Prediction Model WPDINM

According to the weighted PPI network *WppiN*, let *N*_*p_i_*_ and *N_pj_* be the sets of neighboring nodes of *p_i_* and *p*_*j*_, respectively, then *N_pi_*∩*N_pj_* = {*p*_1_,*p*_2_,…,*p*_*T*_} is the set of common neighbors of both *p_i_* and *p_j_*. Supposing that there is *WppiM*(*p*_1_,*p*_*j*_)[*cpsbreak*]≤*WppiM*(*p*_2_,*p*_*j*_)≤…≤*WppiM*(*p*_*T*_,*p*_*j*_), then we define the allocation possibility of weight from *p_i_* to *p_j_* as follows:

(26) $$W\,A\,P\,\left(p_{i},p_{j}\right)=W\,p\,p\,i\,M\,\left(p_{i},p_{T}\right)*W\,p\,p\,i\,M\,\left(p_{T},p_{j}\right)$$

Similarly, supposing that there is *WppiM* (*p*_1_,*p*_*i*_) ≤ *WppiM* (*p*_2_,*p*_*i*_) ≤…≤ *WppiM* (*p*_*T*_,*p*_*i*_), then we define the allocation possibility of weight from *p_j_* to *p_i_* as follows:

(27) $$W\,A\,P\,\left(p_{j},p_{i}\right)=W\,p\,p\,i\,M\,\left(p_{j},p_{T}\right)*W\,p\,p\,i\,M\,\left(p_{T},p_{i}\right)$$

Hence, based on the above formulas, for any two given protein nodes *p_i_* and *p_j_* in *WppiN*, we can obtain an allocation possibility matrix of weights between them as follows:

(28) $$W\,A\,P\,M\,\left(p_{i},p_{j}\right)=\left\{ \begin{array}{c} \rho *W\,A\,P\,\left(p_{i},p_{j}\right)/\sum_{k}W\,A\,P\,\left(p_{i},p_{k}\right):i\,f\,\sum_{k}W\,A\,P\,\left(p_{i},p_{k}\right)\neq 0\\ 0:\;\text{Otherwise} \end{array}\right.$$

Where ρ is the adjustment parameter with a value between 0 and 1.

Based on the above allocation possibility matrix *WAPM*, let a possibility vector *S*_*t+1*_ denote the vector of scores of proteins at the (*t* + 1)*^th^* iteration, then we can calculate the proteins ranks iteratively as follows:

(29) $$S_{t+1}=\mu *W\,A\,P\,M*S_{t}+\left(1-\mu \right)\,S_{0}$$

Where μ ∈ (0,1) is a scale parameter for adjusting the proportion of the current score vector *S_t_* and initial score vector *S_0_*.

Based on the above descriptions, the algorithm WPDINM can be briefly described as follows.

**Algorithm 1:** WPDINM

**Input:** domain data, Original PPI network, original protein-domain network, subcellular data, orthologous data, iterative error value ε, the proportion regulation parameters β,φ,θ,τ,ω,μ

**Output:** proteins score vector *S*

**Step 1**: Establishing weighted PPI network based on formulas (1–7);

**Step 2**: Establishing weighted domain-domain network based on formulas (8, 9);

**Step 3**: Establishing weighted protein-domain network based on formulas (10–12);

**Step 4**: Initializing proteins scores based on formulas (13–25);

**Step 5**: Establishing allocation network based on formulas (26–28);

**Step 6**: Calculating the *S*_*t+1*_based on the formula (29),let *t* = *t*+1;

**Step 7**: Repeating Step 6 until there is ||*S*_*t* + 1_−*S*_*t*_||^2^ < ε;

**Step 8**: Sorting the proteins scores for vector *S*_*t+1*_ through descending order.

## Results

### Comparison of Twelve Essential Proteins Prediction Measures

**FIGURE 2:**
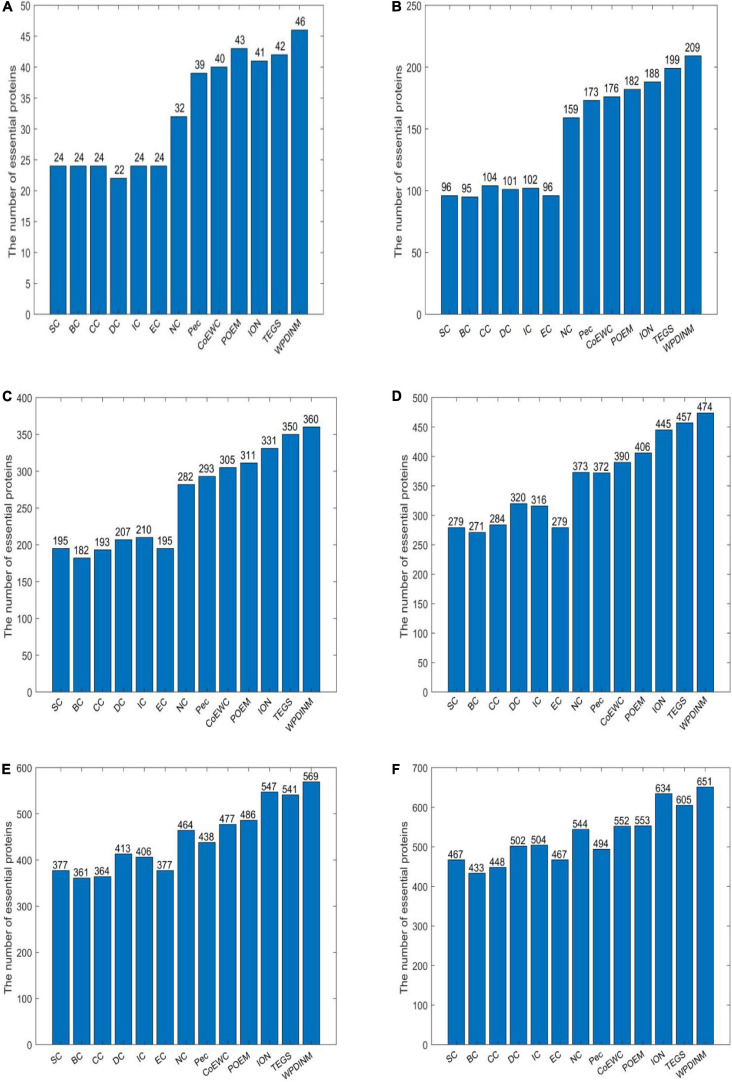
**(A)** Top 1% ranked proteins, **(B)** Top 5% ranked proteins, **(C)** Top 10% ranked proteins, **(D)** Top 15% ranked proteins, **(E)** Top 20% ranked proteins, **(F)** Top 25% ranked proteins. This bar chart shows the comparison of the number of essential proteins predicted by WPDINM and other models, such as SC, BC, CC, DC, IC, EC, NC, Pec, CoEWC, POEM, ION, TEGS based on the DIP database.

The data presented by the bar chart illustrates that the identification performance of WPDINM exceeds the other measures by comparing the forecast accuracy from top 1% to top 25% proteins. It’s apparent from Figure 2 that, with the comparison of prediction accuracy in the top 1% proteins, 90.19% of the true key proteins are detected by the WPDINM method. By deferring the top 5% of proteins, the identification precision of WPDINM is up to 81.96%. The prediction result from the top 10% of proteins shows that the percentage of essential proteins identified by WPDINM is 70.58%. The prediction accuracies of WPDINM are 27.4, 19.6, 15.3, 13.2, 10.3, and 8.4% higher than the NC method from the top 1% to top 25%. By comparing it with the TEGS method, the precision of WPDINM increase by 3.6% from the top 25% of proteins.

**FIGURE 3:**
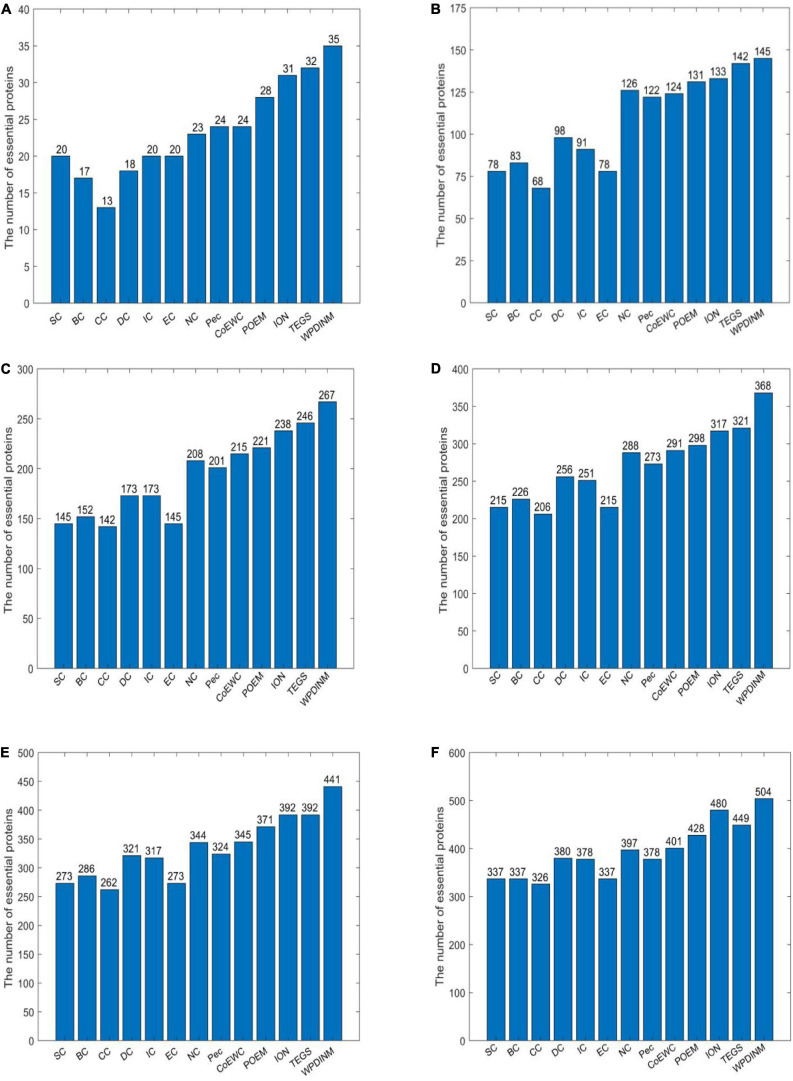
**(A)** Top 1% ranked proteins, **(B)** Top 5% ranked proteins, **(C)** Top 10% ranked proteins, **(D)** Top 15% ranked proteins, **(E)** Top 20% ranked proteins, **(F)** Top 25% ranked proteins. This bar chart shows the comparison of the number of essential proteins predicted by WPDINM and other models, such as SC, BC, CC, DC, IC, EC, NC, Pec, CoEWC, POEM, ION, TEGS based on the Krogan database.

Figure 3 shows the identification accuracy in the Krogan database. By observing the top 1% of proteins, the true essential proteins predicted by WPDINM make up 95%. With the top 5% proteins, 145 essential proteins detected. For the top 10% proteins, the proportion of essential proteins detected by the WPDINM is 5.7% observably higher than TEGS. For the top 15% and top 20% of proteins, the WPDINM can acquire 66.7% and 60% of the identification accuracy. In particular, in the top 25% candidate proteins, the prediction accuracy of WPDINM is, respectively, enhanced by 5.9% by comparing with TEGS. From what has been analyzed above, we can conclude that, whether in the DIP dataset or the Krogan database, the prediction performance of WPDINM is superior to these methods.

### Validated by Jackknife Methodology

**FIGURE 4:**
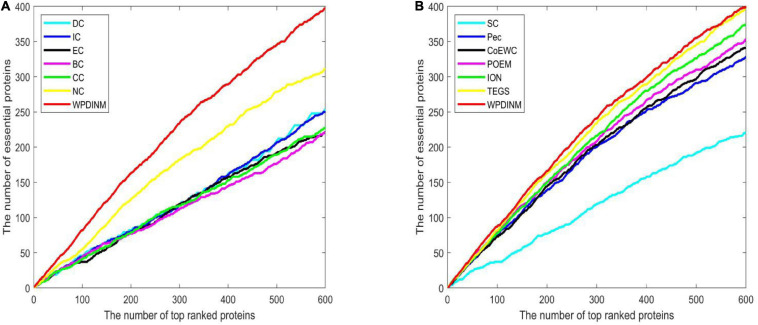
Comparison of Jackknife curves of the WPDINM and 12 kinds of methods based on the DIP dataset. **(A)** Comparison of Jackknife curves of WPDINM and 6 other methods including DC, IC, EC, BC, CC, NC. **(B)** Comparison of Jackknife curves of WPDINM and 6 other measures such as SC, Pec, CoEWC, POEM, ION, TEGS.

To further assess the prediction effect for WPDINM, the Jackknife Methodology is adopted to compare WPDINM with other methods. Figure 4 shows the comparison results from the top 600 ranked proteins in the DIP dataset between the WPDINM method and other methods. As is revealed by Figure 4A, we can see that the WPDINM has more advantages than six prediction methods including IC, DC, CC, NC, BC, and EC. Figure 4B indicates that the performance of WPDINM exceeds the six methods: SC, Pec, CoEWC, POEM, ION, TEGS, respectively.

**FIGURE 5:**
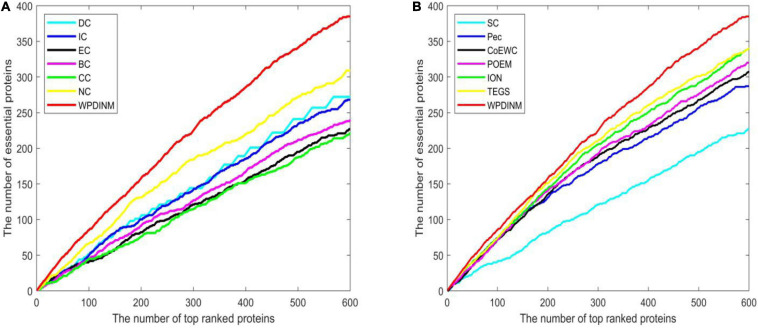
Comparison of Jackknife curves of the WPDINM and 12 kinds of methods based on the Krogan database. **(A)** Comparison of Jackknife curves of WPDINM and 6 other methods including DC, IC, EC, BC, CC, and NC. **(B)** Comparison of Jackknife curves of WPDINM and 6 other measures such as SC, Pec, CoEWC, POEM, ION, TEGS.

Figure 5 indicates the comparison result from the top 600 ranked proteins between the WPDINM and other measures in the Krogan dataset. From Figure 5A, it can be seen that the curve of WPDINM is above the curves of other competitive methods, containing DC, IC, EC, BC, CC, and NC. From Figure 5B, we can observe that the WPDINM is superior to the six methods including SC, Pec, CoEWC, POEM, ION, and TEGS.

### Differences Between WPDINM and Other Methods

To compare the differences between WPDINM and other methods, we select the top 500 ranked proteins to compare the WPDINM with the 11 methods. The results of the comparison are shown in Tables 1, 2. The Methods (Me) is a series of measures compared with the WPDINM methods. WPDINMMe is a set of proteins identified by the WPDINM and one of the Me. |*WPDINMMe*|denotes elements numbers of the WPDINMMe. WPDINM−Me represents the proteins detected by WPDINM except the proteins detected by both WPDINM and one of Me. Similarity, Me−WPDINM denotes the the proteins detected by one of Me except the proteins detected by both WPDINM and one of Me. |*WPDINM*−Me| and |*Me*−*WPDINM*| are the numbers of proteins in WPDINM−Me, Me−WPDINM, respectively. The data provided in Table 1 shows the distinction between the WPDINM method and the eleven kinds of methods in the DIP dataset. It can be found from the second column of the table that the numbers of proteins identified by WPDINM and DC, IC, CC, BC, IC, EC are fewer than 200 proteins. In terms of the data for NC, the numbers of common proteins detected by both WPDINM and NC is just less than half. The proportions of overlapping proteins predicted by WPDINM and Pec, CoEWC, POEM are not more than half. Table 2 reflects the differences of between WPDINM methods and other methods in the Krogan database. From Table 2 we can see that the proportion of key proteins in {WPDINM-Me} is higher than one of the methods.

**TABLE 1:** The common and differences between the WPDINM and the top 500 proteins detected by other methods (DIP).

Methods (Me)	|WPDINM ∩ Me|	|Me-WPDINM|	Percentage of essential proteins in {Me-WPDINM} (%)	|WPDINM-Me|	Percentage of essential proteins in{WPDINM-Me} (%)
DC	168	332	22.89	332	68.98
IC	170	330	23.94	330	68.79
EC	139	361	24.65	361	69.81
SC	139	361	24.65	361	69.81
BC	134	366	21.31	366	69.95
CC	133	367	24.25	367	69.48
NC	225	275	36.36	275	64
Pec	213	287	38.68	287	60.98
CoEWC	226	274	39.05	274	60.22
POEM	243	257	40.86	257	58.37
ION	307	193	41.97	193	56.99
TEGS	265	235	52.76	235	57.02

**TABLE 2:** The common and differences between the WPDINM and the top 500 proteins detected by other methods (Krogan).

Methods (Me)	|WPDINM ∩ Me|	|Me-WPDINM|	Percentage of essential proteins in{Me-WPDINM} (%)	|WPDINM-Me|	Percentage of essential proteins in{WPDINM-Me} (%)
DC	195	305	32.13	305	64.92
IC	196	304	30.59	304	65.79
EC	166	334	25.75	334	69.46
SC	166	334	25.75	334	69.46
BC	169	331	31.12	331	70.09
CC	158	342	23.98	342	69.01
NC	217	283	38.52	283	62.89
Pec	193	307	34.85	307	62.21
CoEWC	199	301	37.21	301	61.46
POEM	211	289	39.10	289	61.59
ION	305	195	36.41	195	61.54
TEGS	226	274	44.52	274	59.12

**FIGURE 6:**
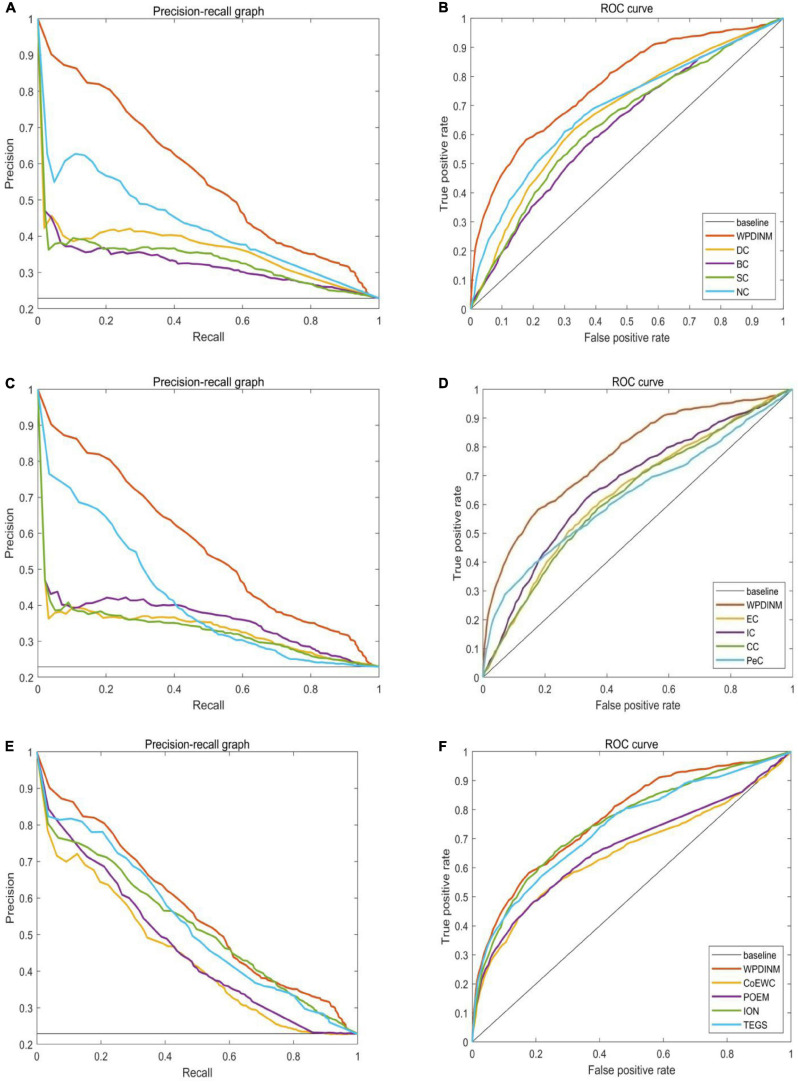
Comparison of PR curves and ROC curves between WPDINM and the other competing methods based on the DIP database. **(A)** The PR curves of DC, BC, SC, NC. **(B)** The ROC curves of DC, BC, SC, NC. **(C)** The PR curves of EC, IC, CC, PeC. **(D)** The ROC curves of EC, IC, CC, PeC. **(E)** The PR curves of CoEWC, POEM, ION, TEGS. **(F)** The ROC curves of CoEWC, POEM, ION, TEGS.

**FIGURE 7:**
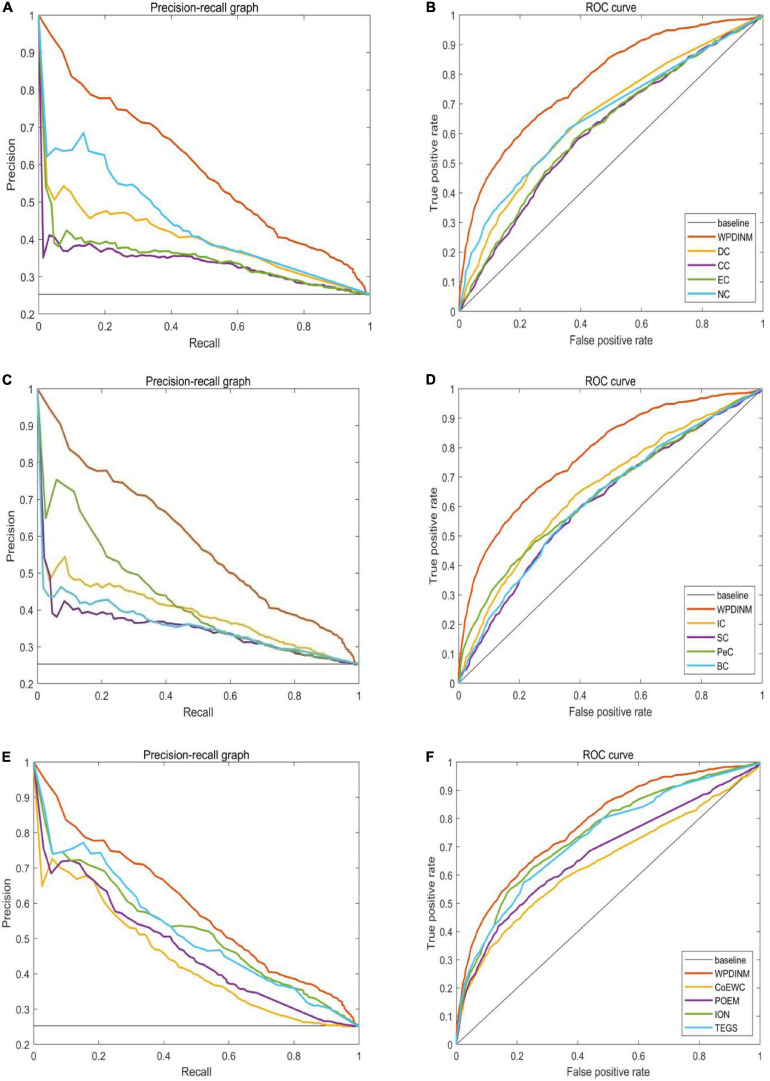
Comparison of PR curves and ROC curves between WPDINM and the other competing methods based on the Krogan database. **(A)** The PR curves of DC, CC, EC, NC. **(B)** The ROC curves of DC, CC, EC, NC. **(C)** The PR curves of IC, SC, PeC, BC. **(D)** The ROC curves of IC, SC, PeC, BC. **(E)** The PR curves of CoEWC, POEM, ION, TEGS. **(F)** The ROC curves of CoEWC, POEM, ION, TEGS.

We further employ the receiver operating characteristic curve (ROC) and Precision-recall curve (PR) to test the prediction ability of the WPDINM model. The larger the area under the ROC curve (AUC), the better the prediction effect of the measure. The AUC data for all methods are collected in Table 3. Figures 6, 7 show the ROC curves and PR curves of the WPDINM method and various methods based on the DIP database and the Krogan database, respectively. As depicted in Figure 6F, although the ROC curves of WPDINM and ION have a little overlap, the AUC of WPDINM from Table 3 is higher than the ION model. Figure 7 shows that the ROC curve of WPDINM is higher than other competitive measures in the Krogan database.

**TABLE 3:** The AUCs of WPDINM and nine different methods in the Krogan database and DIP database.

Method	AUC (DIP)	AUC (Krogan)
DC	0.6704	0.6583
IC	0.6657	0.6573
EC	0.6384	0.6167
BC	0.625	0.6248
SC	0.6384	0.6167
CC	0.6291	0.6114
NC	0.6879	0.6584
Pec	0.6329	0.6316
CoEWC	0.6513	0.6404
POEM	0.6662	0.6726
ION	0.7522	0.7413
TEGS	0.7386	0.7287
WPDINM	0.7714	0.778

As shown in Table 4, when comparing with the other 12 measures, the prediction accuracy of WPDINM is highest from top1% to top 25%. This reveals that the indication effect of the WPDINM model is better than 12 competing methods and that the WPDINM method has applicability to a large extent.

**TABLE 4:** Number of essential proteins identified by WPDINM and 11 methods based on the GAVIN database.

Methods	Top 1% (19)	Top 5% (93)	Top 10% (196)	Top 15% (279)	Top 20% (371)	Top 25% (464)
SC	0	17	87	130	190	240
EC	0	38	94	134	166	209
BC	9	40	85	122	162	201
DC	7	36	101	158	222	264
IC	16	55	119	163	213	254
CC	11	45	93	135	180	221
NC	11	51	123	170	213	259
Pec	15	69	142	193	238	285
CoEWC	16	69	136	190	237	275
POEM	17	74	148	199	249	296
ION	17	73	150	207	263	312
WPDINM	17	83	156	223	281	333

### The Analysis of Parameters

Because the prediction precision needs to be enhanced, we set a proportions parameter μ ∈ (0,1) in iterative formula (29). As is demonstrated in Table 5, we can see that in the DIP dataset, different values of parameter μ can have various influences on the experiment result. The statistics show the prediction accuracy in the top 1% to the top 25% proteins, when the parameter μ is set to a different value. It can be seen that the forecast accuracy slightly fluctuates, with the value of μ increasing. We repeat the same operation in the Krogan database. The data in Table 6 presents the prediction performance from the Krogan database when parameter changing. Finally, because the prediction result is most competitive when the value of μ is 0.4, we choose to compare it with other methods.

**TABLE 5:** Influence of the parameter μ on WPDINM’s predication accuracy (DIP).

μ	0.1	0.2	0.3	0.4	0.5	0.6	0.7	0.8	0.9
**Rank**									
Top 1%	46	46	46	46	46	46	46	46	46
Top 5%	212	211	210	209	209	209	209	210	210
Top 10%	361	360	361	360	359	359	359	359	357
Top 15%	472	471	470	474	476	476	478	478	479
Top 20%	566	568	568	569	568	569	570	570	569
Top 25%	651	651	651	651	651	652	652	652	649

**TABLE 6:** Influence of the parameter μ on WPDINM’s predication accuracy (Krogan).

μ	0.1	0.2	0.3	0.4	0.5	0.6	0.7	0.8	0.9
**Rank**									
Top 1%	35	35	35	35	35	35	35	35	35
Top 5%	147	147	146	145	146	146	145	145	148
Top 10%	268	267	268	267	267	265	264	264	264
Top 15%	369	369	367	368	366	367	369	367	365
Top 20%	440	441	441	441	439	436	437	436	437
Top 25%	500	502	502	504	503	504	504	503	501

For the sake of achieving higher prediction accuracy, we set a series of parameters. When calculating the weighted protein-protein network, we add two parameters β,γ to the computing formula (7). β and γ are adopted to regulate the ratio of two kinds of similarity between proteins. When the values of β and γ are set to 0.5, the WPDINM method obtains the best prediction effect. In formula (19), the parameters φ, θ and τ are employed to adjust the proportion of three features such as subcellular localization, orthologous information, and triangles features. The best experimental result is obtained by setting φ, θ and τ to 0.25, 0.35, and 0.45, respectively. Moreover, in formula (25), when the value of ω is set to 0.7, the WPDINM obtains the best performance.

## Discussion

Essential proteins perform a crucial role in medicine and disease research, which deepen understanding of biological life processes. Accordingly, the prediction of key proteins has become a popular topic in recent years and deserves close attention. Recently, most computational models combining PPI networks and biological information are designed so that simple use of PPI data is unfavorable for achieving prediction accuracy.

The present study proposes a prediction algorithm to detect key proteins by integrating a PPI network and series of protein feature data. Firstly, we construct the weighted PPI network based on the original PPI network and gene expression data processed by DFT. Next, the weighted domain-domain network is established based on the original protein-domain network. Then, by integrating the weighted domain-domain network with the weighted PPI network, the new weighted protein-domain network is further constructed. After that, we assign the initial scores for each protein by combining the topological feature and some biological information such as orthologous information, and subcellular information. Finally, we design a novel iteration algorithm based on the PageRank algorithm to compute protein scores iteratively. As a result, to testify the performance of the WPDINM algorithm, the WPDINM method is applied to three datasets including the DIP database, the Krogan database, and the Gavin database. The experimental result shows that the WPDINM achieves better indication than competitive methods.

## Data Availability Statement

The datasets generated for this study can be found in the online repositories. The names of the repository/repositories and accession number(s) can be found in the article/Supplementary Material.

## Author Contributions

ZM and LW conceived and designed the study. ZM, ZC, and LK obtained and processed datasets. ZM and LK wrote the manuscript. YT, ZZ, and XL provided suggestions and supervised the research. All authors contributed to the article and approved the submitted version.

## Conflict of Interest

The authors declare that the research was conducted in the absence of any commercial or financial relationships that could be construed as a potential conflict of interest. The handling editor QZ declared a past co-authorship/collaboration with one of the authors LW.
